# Understanding Cancer Survivorship Care Needs Using Amazon Reviews: Content Analysis, Algorithm Development, and Validation Study

**DOI:** 10.2196/71102

**Published:** 2025-09-23

**Authors:** Liwei Wang, Qiuhao Lu, Rui Li, Taylor B Harrison, Heling Jia, Ming Huang, Heidi Dowst, Rui Zhang, Hoda Badr, Jungwei W Fan, Hongfang Liu

**Affiliations:** 1 Department of Clinical and Health Informatics, McWilliams School of Biomedical Informatics, The University of Texas Health Science Center at Houston, 7000 Fannin Street, Suite 600, Houston, TX, 77030, United States, 1 713-500-3900; 2 Department of Health Data Science and Artificial Intelligence, McWilliams School of Biomedical Informatics, The University of Texas Health Science Center at Houston, Houston, TX, United States; 3 Department of Artificial Intelligence and Informatics, Mayo Clinic, Rochester, MN, United States; 4 Bioinformatics and Computational Biology, University of Minnesota, Twin Cities, MN, United States; 5 Dan L Duncan Comprehensive Cancer Center, Baylor College of Medicine, Houston, TX, United States; 6 Department of Surgery, University of Minnesota, Twin Cities, MN, United States; 7 Department of Medical Oncology, Thomas Jefferson University, Philadelphia, PA, United States

**Keywords:** real-world data, cancer research, cancer survivorship care, natural language processing, annotation, baseline models, deep learning, large language model

## Abstract

**Background:**

Complementary therapies are being increasingly used by cancer survivors. As a channel for customers to share their feelings, outcomes, and perceived knowledge about the products purchased from e-commerce platforms, Amazon consumer reviews are a valuable real-world data source for understanding cancer survivorship care needs.

**Objective:**

In this study, we aimed to highlight the potential of using Amazon consumer reviews as a novel source for identifying cancer survivorship care needs, particularly related to symptom self-management. Specifically, we present a publicly available, manually annotated corpus derived from Amazon reviews of health-related products and develop baseline natural language processing models using deep learning and large language model (LLM) to demonstrate the usability of this dataset.

**Methods:**

We preprocessed the Amazon review dataset to identify sentences with cancer mentions through a rule-based method and conducted content analysis including text feature analysis, sentiment analysis, topic modeling, cancer type, and symptom association analysis. We then designed an annotation guideline, targeting survivorship-relevant constructs. A total of 159 reviews were annotated, and baseline models were developed based on deep learning and large language model (LLM) for named entity recognition and text classification tasks.

**Results:**

A total of 4703 sentences containing positive cancer mentions were identified, drawn from 3349 reviews associated with 2589 distinct products. The identified topics through topic modeling revealed meaningful insights into cancer symptom management and survivorship experiences. Examples included discussions of green tea use during chemotherapy, cancer prevention strategies, and product recommendations for breast cancer. Top 15 symptoms in reviews were also identified, with pain being the most frequent symptom, followed by inflammation, fatigue, etc. The annotation labels were designed to capture cancer types, indicated symptoms, and symptom management outcomes. The resulting annotation corpus contains 2067 labels from 159 Amazon reviews. It is publicly accessible, together with the annotation guideline through the Open Health Natural Language Processing (OHNLP) GitHub. Our baseline model, Bert-base-cased, achieved the highest weighted average *F*_1_-score, that is, 66.92%, for named entity recognition, and LLM gpt4-1106-preview-chat achieved the highest *F*_1_-score for text classification tasks, that is, 66.67% for “Harmful outcome,” 88.46% for “Favorable outcome” and 73.33% for “Ambiguous outcome.”

**Conclusions:**

Our results demonstrate the potential of Amazon consumer reviews as a novel data source for identifying persistent symptoms, concerns, and self-management strategies among cancer survivors. This corpus, along with the baseline natural language processing models developed for named entity recognition and text classification, lays the groundwork for future methodological advancements in cancer survivorship research. Importantly, insights from this study could be evaluated against established clinical guidelines for symptom management in cancer survivorship care. By revealing the feasibility of using consumer-generated data for mining survivorship-related experiences, this study offers a promising foundation for future research and argumentation analysis aimed at improving long-term outcomes and support for cancer survivors.

## Introduction

The treatment of cancer results in unintended side effects and outcomes including pain, fatigue, weakness, anorexia, constipation, anxiety, dyspnea, nausea, and vomiting. These symptoms may emerge during active treatment and frequently persist into the posttreatment phase, necessitating continued monitoring and support. The National Cancer Institute defines cancer survivorship care as beginning at cancer diagnosis and continuing through the remainder of a patient’s life, encompassing both medical and supportive care needs across the continuum of care [1]. Recognizing its importance, the Institute of Medicine, the National Cancer Institute, and the American Society of Clinical Oncologists have increasingly prioritized survivorship care as a critical component of efforts to improve long-term cancer outcomes and quality of life [2-4].

Cancer survivorship care extends well beyond surveillance for recurrence or secondary malignancies. According to the National Cancer Institute’s National Standards for Cancer Survivorship Care, high-quality survivorship care should address several key focus areas: communication and coordination of care, prevention and surveillance of new or recurrent cancers, symptom management and supportive care, and provision of practical resources to help survivors navigate life after treatment [2]. These standards underscore the need for a comprehensive, patient-centered approach that supports cancer survivors during and after the transition out of active oncology care. Addressing ongoing needs—such as managing long-term and late effects of treatment, promoting healthy behaviors, and ensuring access to psychosocial support—is critical to optimizing survivors’ quality of life, particularly during periods of reduced clinical oversight.

Digital platforms such as social media and forums have emerged as important spaces where cancer survivors seek support, share experiences, and access health information [5,6]. These platforms also offer a rich, untapped source of real-world data for researchers seeking to understand the survivorship experience from the patient’s perspective. Advances in large learning models such as OpenAI, Gemini, and LLAMA have also made it increasingly feasible to process and extract insights from these large, unstructured text datasets.

One such promising resource is Amazon’s consumer product review system. As a widely used e-commerce platform with national reach—including rural and underserved areas—Amazon provides consumers with the opportunity to share detailed reflections on their experiences with health and wellness products. These reviews often contain personal narratives about symptom self-management, perceived product effectiveness, and emotional responses, which may be particularly relevant for understanding the needs of cancer survivors. Theoretically, language content (eg, the percentage of words related to a topic)—such as the proportion of words associated with specific symptoms or outcomes—can reflect an individual’s focus and meaning-making processes [7]. Analyzing large-scale consumer-review data can thus offer insights into consumer knowledge, attitudes, and behaviors from a population health perspective [8,9]. Furthermore, the structured format of Amazon reviews, coupled with the inclusion of product use experiences and verified purchase indicators, enhances their value as a real-world data source for health care studies.

To process the large volume of unstructured text generated by consumers, text mining and natural language processing (NLP) techniques are essential for extracting meaningful patterns and insights. For example, previous applications of NLP in health forums have successfully extracted clinically relevant information such as treatment types, medication names, and side effects from cancer-related user-generated content [10]. Health NLP—an interdisciplinary field that integrates computational linguistics with health care—has received growing attention in recent years [11], leading to the development of a range of NLP tools and systems. One such platform, Open Health NLP (OHNLP), provides open-source clinical NLP software that facilitates large-scale analysis of free-text health data [12-14].

Despite the growing application of NLP in health care, existing studies using Amazon reviews as data sources for health-related insights have largely focused on noncancer domains such as erectile dysfunction and testosterone supplements [15], eye health [16], and chronic pain [17]. These studies demonstrate the feasibility of extracting product-related health experiences from consumer reviews. They also underscore a critical gap: there has been limited exploration of how individuals affected by cancer—particularly survivors—use Amazon to share experiences with complementary therapies, long-term symptoms, and navigate posttreatment concerns. Understanding these patterns is crucial for addressing the informational and self-management needs of cancer survivors.

Amazon product reviews represent a novel and largely untapped data resource for exploring cancer symptom management from the survivor’s perspective. These reviews may reveal implicit information about how survivors respond to persistent symptoms, evaluate over-the-counter and complementary therapies, and seek support outside of traditional health care settings. Annotated corpora are critical for training and evaluating NLP algorithms that can reliably identify these patterns. However, existing Amazon review datasets have been developed primarily for sentiment analysis [18-20], and there is a lack of manually annotated cancer-specific corpora that focus on survivorship-related constructs.

In this study, we aim to address this gap by evaluating the potential of Amazon consumer reviews to surface cancer survivors’ ongoing concerns, persistent symptoms, and unmet needs. Specifically, we (1) present a publicly available, manually annotated corpus derived from Amazon reviews of health-related products, and (2) develop baseline NLP models using deep learning and large language model (LLM) approaches to demonstrate the usability of this dataset. These tools provide the foundation for future research that leverages consumer-generated data to inform survivorship interventions and improve long-term outcomes for cancer survivors.

## Methods

### Data Source

We used the preprocessed dataset of Health & Personal Care category containing reviews and metadata from Amazon between May 1996-July 2014 [21]. This dataset has been deduplicated, consisting of 2,982,326 reviews and 263,032 metadata. Review data includes reviewer ID, the Amazon Standard Identification Number (ASIN) which Amazon uses to identify products, reviewer name, helpfulness of rating, review text, overall rating (1‐5 stars), summary of review, and review time. Metadata of the reviews includes ASIN, title, price, image URL, what items the customer also bought, what items the customer also viewed, what items the customer bought together, sales rank, brand, and categories. ASIN is the primary key to link review text and metadata.

### Study Design

**Figure 1.**
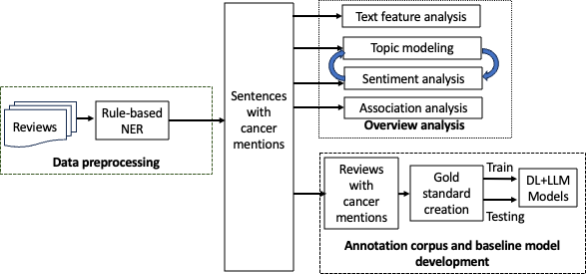
Study design. DL: deep learning; LLM: large language models; NER: named entity recognition.

Figure 1 shows the study design. Multiple methodologies have been developed to identify named entities in texts, that is, machine learning, deep learning, hybrid, and rule-based methods [22]. In the first step, we used a rule-based method to identify a set of review texts with cancer mentions for a high-level content analysis. We then created an annotated corpus from the set of review texts and developed baseline NLP models, including deep learning and LLM, for named entity recognition (NER) and text classification.

### Data Preprocessing With Rule-Based NLP Method

To identify the reviews with cancer mentions, we prepared a cancer dictionary based on the cancer branch of the Disease Ontology. It includes cell type cancer and organ system cancer integrated from different terminologies and vocabularies including the Catalog of Somatic Mutations in Cancer, The Cancer Genome Atlas, International Cancer Genome Consortium, Therapeutically Applicable Research to Generate Effective Treatments, Integrative Oncogenomics, and the Early Detection Research Network [23]. In total, there are 4343 cancer term variants corresponding to 1535 cancer concepts. The cancer terms were prepared into the symbolic lexicon format compatible with the Open Health Natural Language Processing (OHNLP) Toolkit’s NLP engine MedTagger [24]. The open-source clinical NLP pipeline analyzed review texts and identified cancer-related medical concepts along with the assertion status of the cancer concept including certainty (ie, positive, negative, and hypothetical and possible). We kept only positive cancer concept mentions for further analysis.

### Content Analysis

We summarized the features of texts containing sentences with cancer mentions, conducted sentiment analysis, topic modeling, and visualization of cancer types and symptoms association for the review sentences with cancer mentions to gain insights into the prevailing themes and mood surrounding discussions related to cancer within the dataset.

#### Text Feature Analysis

To understand the text features of review data, we performed text complexity analysis to summarize review texts containing the sentences with cancer mention, including number of review texts, number of sentences, and number of words. For comparison purposes, the above metrics were also calculated for the entire collection of reviews from the Health & Personal Care category.

#### Sentiment Analysis

Bert-base-multilingual-uncased-sentiment [25] is a fine-tuned model from a bertbase-multilingual-uncased model for sentiment analysis on product reviews in 6 languages including English. Based on 5000 held-out product reviews for English, the accuracy (exact), that is, exact match for the number of stars is 67%. Accuracy (off-by-1), that is, the percentage of reviews where the number of stars the model predicts differs by a maximum of 1 from the number given by the human reviewer is 95%. The fine-tuned model was used for sentiment analysis of review sentences with cancer term mentions. This model predicts the sentiment of input text as a number of stars (between 1 and 5). The higher the sentiment score, the more positive the review. The lack of context has been one major challenge in sentiment analysis that can affect the interpretation of sentiment [26]. We consider that identifying customer attitudes based on the sentence containing cancer mentions instead of the whole review text can be better constructive in understanding consumers’ efficacy and safety perceptions. The sentiment of the review sentences with cancer mentions detected by Medtagger was further analyzed to identify positive or negative attitudes toward the product [27,28]. We analyzed the distribution of sentiment scores across the review sentences with cancer mentions, and the trend of average sentiment score between 1996 and 2014.

#### Topic Modeling

We used a sentence embedding model (ie, bge-small-en) [29] to transform the textual content of reviews into numerical embeddings. These embeddings capture the semantic essence of each document in a high-dimensional space. We then applied UMAP (Uniform Manifold Approximation and Projection) [30] to the embeddings for dimensionality reduction. This step is crucial for visualization, as it converts high-dimensional data into a 2-dimensional format suitable for plotting. The core of the analysis is performed by BERTopic [31], a model that identifies distinct topics within the text data. BERTopic relies on sub-models for embeddings (provided by SentenceTransformer of bge-small-en), dimensionality reduction (UMAP), and hierarchical clustering (HDBSCAN) [32]. In addition, a quantized LLM (ie, openhermes-2.5-mistral-7b) [33] is incorporated for topic label generation. After fitting the data to the BERTopic model, topics are extracted along with their probabilities. Each topic is then assigned a label generated by the LLM based on a predefined prompt. These labels are designed to be concise, with a maximum of 5 words, and describe the essence of the documents within each topic.

The chosen sentences were preprocessed by removing stop-words, special characters, and numbers and removing sentences with pets (dog, cat, etc). We detected topics based on all sentences with cancer mentions, as well as the sentences from 5 sentiment score groups. We then visualized the results.

#### Cancer Type and Symptom Association

To explore the relationship between reported cancer types and symptoms, we constructed bipartite graphs based on co-occurrence patterns in the chosen sentences. Symptom mentions were identified using a state-of-the-art LLM for NER, specifically, the UniversalNER-7b-all model, which was applied via a 0-shot strategy. We then calculated the frequency of cancer type-symptom co-occurrences to generate a set of unique pairs. Each bipartite graph consisted of 2 node sets—cancer and symptoms—with edges indicating their association frequency. Node placement was optimized to ensure even distribution and visual clarity within each group. Edge widths were normalized and scaled to reflect the relative frequency of each cancer type-symptom pair, allowing for a visual representation of the strength of association between nodes.

### Development of Gold Standards and Baseline Models

#### Gold Standard Creation

We developed an annotation guideline (Multimedia Appendix 1) to support the systematic labeling of target data elements and their associated class and type designations from Amazon customer reviews. The guideline was designed to be concise in order to minimize annotators’ cognitive load while ensuring consistency and enabling the annotated dataset’s future use for information extraction tasks. The schema of annotated labels is presented in Table S1 in Multimedia Appendix 2. Target concepts included cancer type, indicated symptoms, favorable outcome, harmful outcome, and product, with each concept having class or type options.

The cancer type concept has the class of either human or pet. The indicated symptoms, favorable outcome, and harmful outcome concepts were further categorized as either cancer-related or other, while the product concept was labeled as itself or other. In addition to class and type assignments, we also annotated each cancer type, indicated symptom, favorable outcome, and harmful outcome instance with one of 4 levels of certainty: positive, negative, hypothetical, or possible. For example, in the sentence: “I’ve had salivary gland cancer,” the phrase “salivary gland cancer” was annotated as the cancer type concept, with the human class and positive certainty. In contrast, the phrase “might prevent cancer” in the sentence: “Some people say it might prevent cancer,” was annotated as a favorable outcome concept, with the cancer-related class and hypothetical certainty.

MedTator [13], a free and open-source annotation tool, was used to perform the annotation task. Two annotators with backgrounds in medicine and informatics were first trained to annotate following the annotation guideline. After initial training, inter-annotator agreement (IAA) was assessed during the process. Once the annotators achieved a high level of agreement (*F*_1_-score ≥0.9), they proceeded to independently annotate the review texts. Discrepancies were resolved through an adjudication process involving discussion and consensus, resulting in a finalized gold standard corpus.

A total of 200 review texts containing cancer-related mentions, identified from the first step, were randomly selected for annotation. During the annotation process, we focused on reviews reflecting customer perspectives and excluded those summarizing books or other nonproduct-related content. This yielded a final sample of 159 consumer-generated reviews that were chosen for annotation.

#### Development of Baseline Models

The annotated dataset was used to develop baseline models for 2 NLP tasks: NER and text classification. The goal of the NER task was to identify and classify entities mentioned in consumer reviews, specifically focusing on cancer types, indicated symptoms, and product mentions. For model development, we restricted annotations to human cancer types and cancer-related symptoms. The cancer type entity category included specific diagnoses such as “breast cancer,” “leukemia,” “lymphoma,” and “melanoma,” and only entities annotated with a positive certainty value were included. The indicated symptoms category captured phrases that suggested the condition or symptom the product was used to address (eg, “affected her eye” in the sentence “She had cancer that affected her eye”). The product entity included both direct product mentions and anaphoric references (eg, “this”).

For the text classification task, review excerpts were categorized into one of 3 outcome classes based on product impact on cancer-related conditions: favorable, harmful, or ambiguous. Favorable outcomes are comments where the product is noted to positively affect a cancer-related condition. Harmful outcomes are comments indicating a negative impact on cancer-related conditions. Ambiguous outcomes include comments with possible and hypothetical certainties, reflecting the speculative nature of the feedback.

We developed 2 types of baseline models for the NER and text classification tasks. The first used supervised fine-tuning (SFT) of BERT-like models, with 2 different classification heads on top, that is, token classification and sequence classification, respectively. We evaluated the performance of 2 widely used BERT-like models: bert-base-cased and Bio_ClinicalBERT. The second type of baseline was based on an LLM approach using the gpt4-1106-preview-chat model. We prompted the model to perform both tasks under varying in-context learning conditions: zero-shot, few-shot (using 5 examples), and many-shot (using all available training examples) [34]. The prompts used for NER and text classification are included in Appendix File 3. For both NER and text classification tasks, we partitioned the annotated dataset using an 80-20 train-test split, with 80% of the data used for training and 20% reserved for evaluation.

### Ethical Considerations

The data used in the study were publicly available [21]. As Amazon customer reviews typically do not contain personally identifiable information and we used the public dataset; therefore, personally identifiable information was not a concern in this study.

## Results

### Content Analysis

A total of 4703 sentences containing positive cancer mentions were identified, drawn from 3349 reviews associated with 2589 distinct products. These cancer-related reviews contained a total of 26,078 sentences and 500,087 words, with an average length of 149.3 words per review. For comparison, the broader Health & Personal Care category comprised 2,982,326 reviews, totaling 10,469,336 sentences and 199,501,964 words, with an average of 66.9 words per review. Table 1 shows the distribution of product categories with reviews that include cancer-related mentions.

Table S2 in Multimedia Appendix 2 shows the distribution of sentiment scores across the review sentences with cancer mentions, where scores 1 and 5 prevailed as the top sentiments. Temporarily, there were increasing trends for the average sentiment score of the sentences with cancer mentions from 2004 to 2014 before and after a dip around 2008.

**Table 1.** Distribution of product categories in review sentences of cancer mentions.

Product categories	Number of review sentences with cancer mentions
Health & personal care	4703
Vitamins & dietary supplements	2758
Health care	675
Personal care	361
Sports nutrition	283
Medical supplies & equipment	268
Household supplies	99
Sexual wellness	45

**Figure 2.**
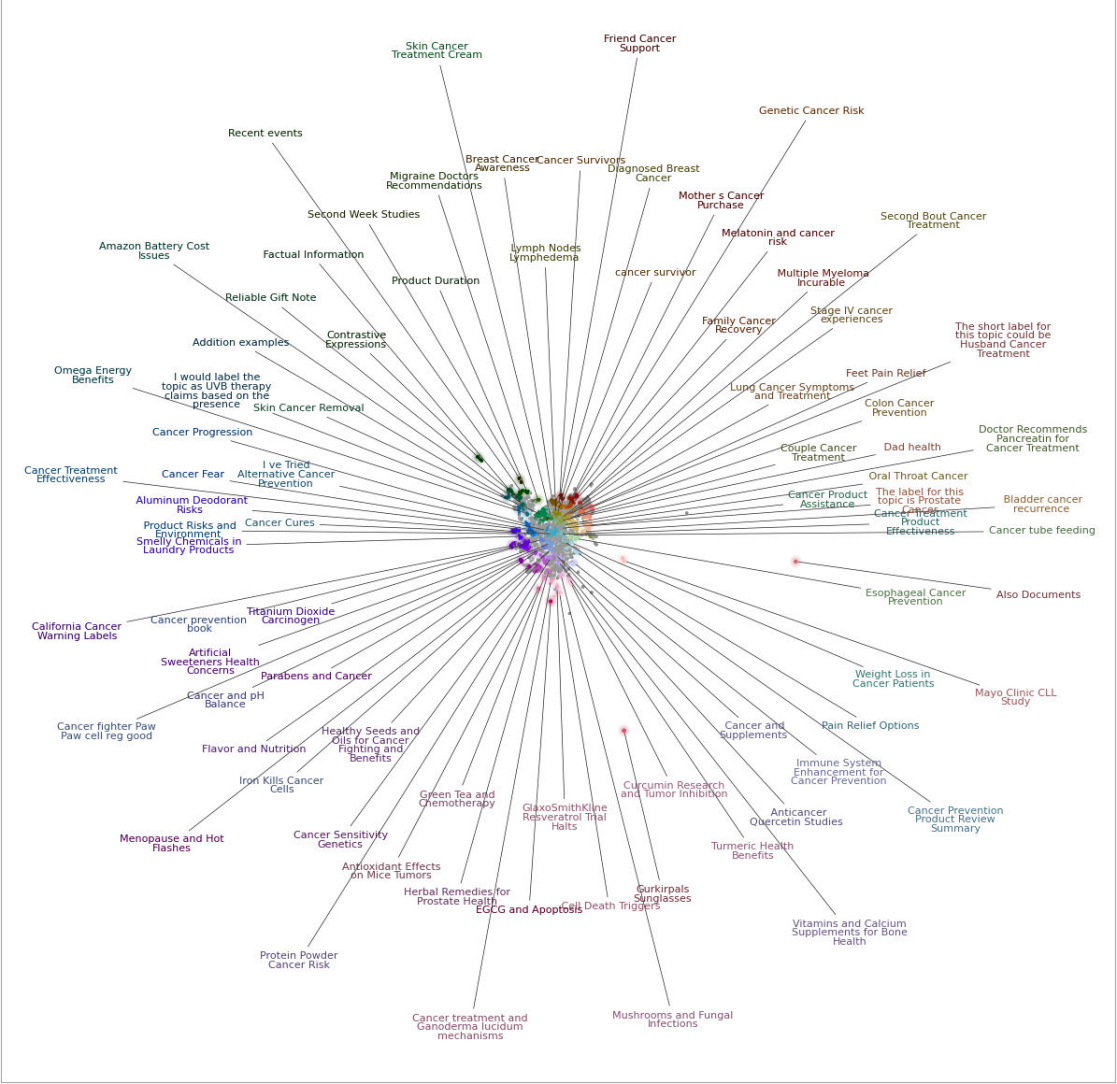
Topic modeling with BERTopic based on all sentences with cancer mentions.

**Figure 3.**
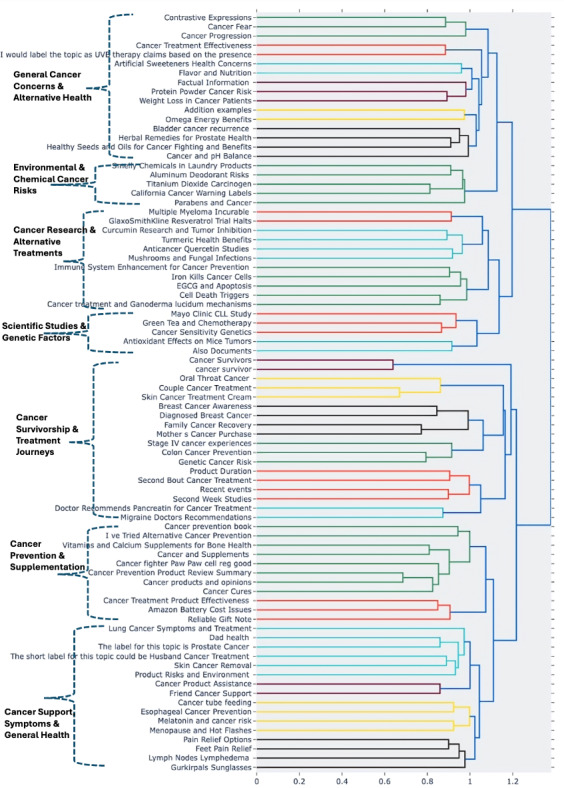
Hierarchical clustering of topics based on all sentences with cancer mentions.

Figure 2 shows the results of topic modeling applied to all sentences containing cancer mentions extracted using the dictionary method. The identified topics revealed meaningful insights into cancer symptom management and survivorship experiences. Examples included discussions of green tea use during chemotherapy, cancer prevention strategies, product recommendations for breast cancer; post-treatment oral health issues, and antioxidant effects on tumor vasculature. To further examine the thematic structure, we conducted hierarchical clustering of these topics, as shown in Figure 3. Cluster labels were generated using GPT-4o (prompting instructions are shown in Multimedia Appendix 3), resulting in the following high-level themes: General Cancer Concerns & Alternative Health; Environmental & Chemical Cancer Risks; Cancer Research & Alternative Treatments; Scientific Studies & Genetic Factors; Cancer Survivorship & Treatment Journeys; Cancer Prevention & Supplementation and Cancer Support, Symptoms & General Health. For example, the General Cancer Concerns & Alternative Health cluster includes discussions related to cancer-related fears and disease progression, while the Cancer Support, Symptoms & General Health cluster captures narratives related to pain management, lymph node involvement, and lymphedema. To quantify the distribution of content across these clusters, we further prompted GPT-4o to assign each sentence derived from topic modeling into one of the 7 clusters using an in-context prompt (Multimedia Appendix 3). As shown in Table S3 in Multimedia Appendix 2, the cluster Cancer Support, Symptoms & General Health accounted for the largest proportion of sentences, followed by Cancer Prevention & Supplementation.

Figures S1-S6 in Multimedia Appendix 4 display the results of hierarchical clustering and topic modeling conducted separately for review sentences grouped by sentiment score 1, 2, 3, 4, and 5. This stratified analysis revealed notable differences in thematic content across sentiment groups. For example, sentiment score group 1 (most negative) contained a higher concentration of topics related to cancer risks, including concerns about carcinogenic ingredients, California Proposition 65 warning labels, artificial sweeteners, product safety, and general ingredient toxicity. In contrast, sentiment score group 5 (most positive) featured a greater number of topics highlighting perceived benefits for cancer-related conditions. These included the purported benefits of iodine for thyroid health, calcium vitamin supplementation for bone health, flaxseed as a complementary therapy, narratives of cancer survivorship and thriving, use of sleep aids during cancer treatment, and various anticancer supplements used by survivors.

**Figure 4.**
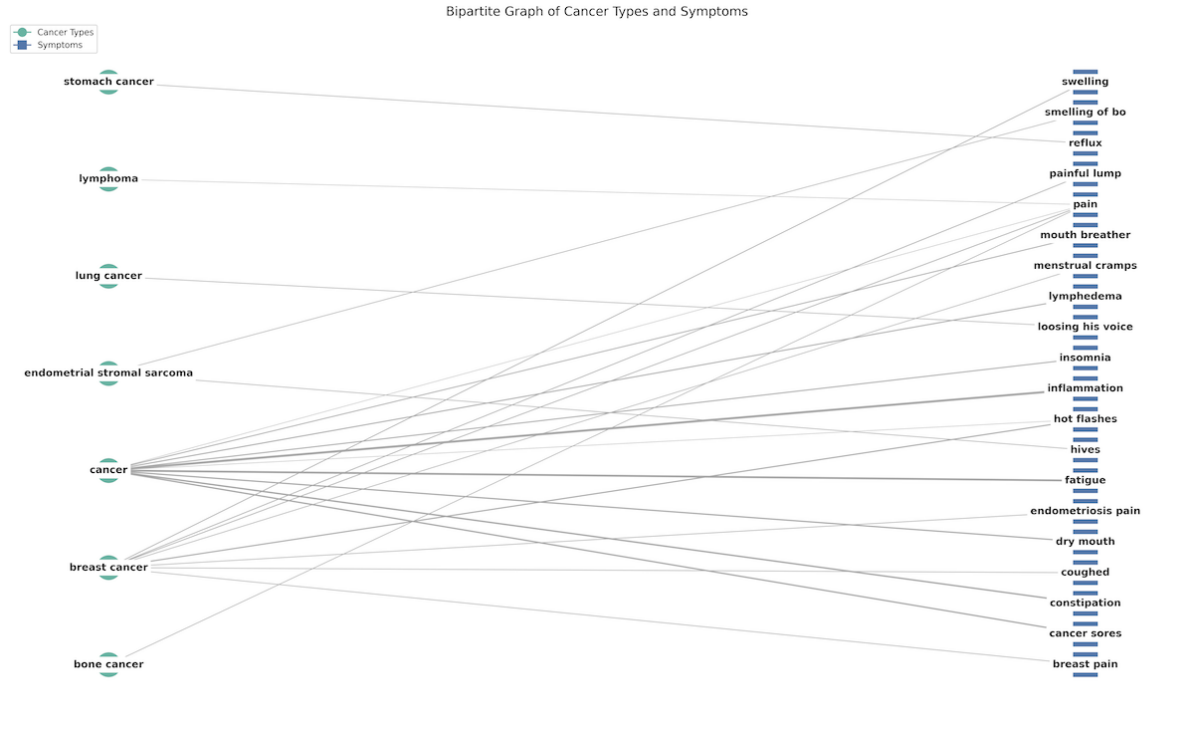
Bipartite graph of cancer types and symptoms extracted by large language model.

Figure 4 shows the bipartite graphs of cancer types with symptoms. The bipartite graph is used to show the association between cancer types and symptoms instead of causal relations in a sentence. The edge between cancer types and symptoms represented the frequency of the cancer type-symptom pairs, showing the strength of each association. Zero-shot LLM extracted detailed symptoms, such as pain, inflammation, fatigue, constipation, etc. Results showed associations between stomach cancer and reflux, breast cancer and menstrual cramps, bone cancer and pain, etc.

Top 15 symptoms in reviews were also identified, with pain being the most frequent symptom, followed by inflammation, fatigue, hot flashes, dry mouth, constipation, cancer sores, nausea, insomnia, neuropathy, lymphedema, incontinence, diarrhea, bloating, fever, and night sweats.

### Annotation Corpus

Table 2 shows the statistics for the resulting annotated corpus for each concept and associated classes (type) and certainties. In total, 2067 labels were generated from 159 reviews. Table S4 in Multimedia Appendix 2 shows the inter-annotator agreements for each concept annotation, with the overall inter-annotator agreement being 0.86. IAA for cancer type is the highest (0.97), and harmful outcome is the lowest (0.63). The annotated corpus is publicly accessible through the OHNLP Github [13,35].

**Table 2.** Statistics of the resulting annotated corpus.

Concepts and class (type)^a^	Certainty			
	Positive	Negative	Hypothetical	Possible
Human				
Cancer_type	131	9	100	2
Pet				
Cancer_type	18	0	3	0
Cancer_related				
Indicated_symptom	105	1	1	0
Harmful_outcome	16	0	5	0
Favorable_outcome	145	0	51	1
Other				
Indicated_symptom	80	0	2	0
Harmful_outcome	23	0	1	0
Favorable_outcome	242	0	15	0

a There were 1,015 labels for Product (itself) and 98 for Product (other).

### Baseline Model Development

In our study, the annotated data is used for two distinctive NLP tasks, ie, named entity recognition and text classification. The dataset for the NER task included 1054 annotated samples, with 80% (843 samples) used for training the model and 20% (211 samples) designated for testing its accuracy. For text classification, the dataset consists of 218 annotated samples, with 80% (174 samples) allocated for training the model and 20% (44 samples) reserved for testing its accuracy. Table 3 shows the statistics of annotation entity labels for model development.

Table 4 shows the performance of Bert-base-cased, Bio_ClinicalBERT, and gpt4-1106-preview-chat in NER. In general, bert-like models outperformed LLM, with 0.6692 weighted average *F*_1_-score for bert-base-cased, 0.6558 for Bio_ClinicalBERT, and the best performance of gpt4-1106-preview-chat was 0.5077 weighted average *F*_1_-score through many-shot strategy. Among the 3 entities, “indicated symptom” showed consistent lower performance across all baseline models compared with the other two entities, that is, “cancer type” and “product,” implying the difficulty of extracting this entity.

Table 5 shows the performance of baseline models in text classification. The performance of LLM gpt4-1106-preview-chat using many-shot strategy exceeded bert-like models. Specifically, the performance of bert-base-cased and Bio_ClinicalBERT in classifying “Harmful outcome” was zero. This could be explained by the limited number, that is, 16, of “Harmful outcome” labels in the gold standard. In addition, the IAA of harmful outcome is the lowest during annotation, implying that “Harmful outcome” classification is the most difficult classification task among all. In contrast, LLM excelled in the scenario of the limited labels, achieving the highest *F*_1_-score for the 3 classes, that is, 0.6667 for “Harmful outcome,” 0.8846 for “Favorable outcome,” and 0.7333 for “Ambiguous outcome.”

**Table 3.** Statistics of annotation entity labels for model development.

Task and target	Criteria	Number label
NER^a^		
Cancer_type	Human, positive	131
Indicated_symptom	Cancer_related, positive	105
Product	Itself	1015
Text classification		
Favorable_outcome	Cancer_related, positive	145
Harmful_outcome	Cancer_related, positive	16
Ambiguous_outcome	Cancer_related, hypothetical and possible	57

a NER: named entity recognition.

**Table 4.** Performance of baseline models in named entity recognition.

Model, learning strategy, and entity	Precision	Recall	*F*_1_-score	Standard error (*F*_1_)	Lower CI (*F*_1_)	Upper CI (*F*_1_)
Bert-base-cased						
SFT^a^						
Cancer_type	0.5366	0.6286	0.5789	0.0493	0.4821	0.6756
Indicated_symptom	0.1667	0.1429	0.1538	0.0360	0.08	0.2245
Product	0.6773	0.7161	0.6962	0.0459	0.6060	0.7863
Micro average	0.6514	0.6905	0.6704	0.047	0.5782	0.7625
Macro average	0.4602	0.4959	0.4763	0.0499	0.3784	0.5741
Weighted average	0.6495	0.6905	0.6692	0.0470	0.5769	0.7614
Bio_ClinicalBERT						
SFT						
Cancer_type	0.5349	0.697	0.6053	0.0489	0.5094	0.7011
Indicated_symptom	0.3000	0.2143	0.2500	0.0433	0.1651	0.3348
Product	0.695	0.6583	0.6762	0.0468	0.5844	0.7679
Micro average	0.6675	0.6462	0.6567	0.0474	0.5636	0.75
Macro average	0.5100	0.5232	0.5105	0.0499	0.4125	0.6084
Weighted average	0.6684	0.6462	0.6558	0.0475	0.5626	0.7489
Zero-shot						
Cancer_type	0.2885	0.6818	0.4054	0.0490	0.3091	0.5016
Indicated_symptom	0.0759	0.4615	0.1304	0.0336	0.06	0.1964
Product	0.3529	0.3243	0.338	0.0473	0.2452	0.4307
Micro average	0.2776	0.3619	0.3142	0.0464	0.2232	0.4051
Macro average	0.2391	0.4892	0.2913	0.0454	0.2022	0.3803
Weighted average	0.3334	0.3619	0.3333	0.0471	0.2409	0.4256
gpt4-1106-preview-chat						
Few-shot						
Cancer_type	0.3148	0.7727	0.4474	0.0497	0.3499	0.5448
Indicated_symptom	0.0536	0.2308	0.087	0.0281	0.03	0.1422
Product	0.4743	0.5405	0.5053	0.0499	0.4073	0.6032
Micro average	0.3857	0.5447	0.4516	0.0498	0.3540	0.5491
Macro average	0.2809	0.5147	0.3465	0.0476	0.2532	0.4397
Weighted average	0.4394	0.5447	0.4791	0.0499	0.3811	0.5770
Many-shot						
Cancer_type	0.4000	0.6364	0.4912	0.05	0.3932	0.589
Indicated_symptom	0	0	0	0	0	0
Product	0.5672	0.5135	0.539	0.0498	0.44	0.6367
Micro average	0.5079	0.4981	0.5029	0.05	0.4049	0.601
Macro average	0.3224	0.3833	0.3434	0.0474	0.2503	0.4364
Weighted average	0.5242	0.4981	0.5077	0.0499	0.4097	0.6056

a SFT: supervised fine-tuning.

**Table 5.** Performance of baseline models in text classification.

Model, learning strategy, and sentiment	Precision	Recall	*F*_1_-score	Standard error (F1)	Lower CI (F1)	Upper CI (F1)
Bert-base-cased						
SFT^a^						
Harmful_outcome	0	0	0	0	0	0
Favorable_outcome	0.6470	0.8800	0.7457	0.0435	0.6603	0.8310
Ambiguous_outcome	0.7000	0.4375	0.5384	0.0498	0.4406	0.6361
Bio_ClinicalBERT						
SFT						
Harmful_outcome	0	0	0	0	0	0
Favorable_outcome	0.6486	0.96	0.7741	0.0418	0.6921	0.8560
Ambiguous_outcome	0.8571	0.375	0.5217	0.0499	0.4237	0.6196
gpt4-1106-preview-chat						
Zero-shot						
Harmful_outcome	0.6667	0.6667	0.6667	0.0471	0.5743	0.7590
Favorable_outcome	0.7368	0.56	0.6364	0.0481	0.5421	0.7306
Ambiguous_outcome	0.4545	0.625	0.5263	0.0499	0.4284	0.6241
Few-shot						
Harmful_outcome	0.5	0.6667	0.5714	0.0494	0.4744	0.6683
Favorable_outcome	0.6429	0.72	0.6792	0.0466	0.5877	0.7706
Ambiguous_outcome	0.3333	0.25	0.2857	0.0451	0.1971	0.3742
Many-shot						
Harmful_outcome	0.6667	0.6667	0.6667	0.0471	0.5743	0.7590
Favorable_outcome	0.8519	0.92	0.8846	0.0319	0.8219	0.9472
Ambiguous_outcome	0.7857	0.6875	0.7333	0.0442	0.6466	0.8199

a SFT: supervised fine-tuning.

## Discussion

### Principal Findings

Complementary therapies are increasingly used by cancer survivors to manage persistent symptoms and long-term side effects. Among breast cancer patients, for example, dietary supplement use has been reported in 67% to 87% of cases [36,37]. However, complementary therapies are often not integrated into routine oncology care, and clinical research evaluating their effectiveness remains limited. One contributing factor is that many patients do not disclose their use of such therapies to providers, creating a significant gap in understanding how survivors self-manage their health outside clinical settings [38,39].

In this study, we explored the potential of Amazon consumer reviews as a novel data source for capturing survivor-reported experiences with symptom management and complementary therapy use. Through content analysis, we identified several dimensions of cancer survivorship care reflected in these reviews.

First, topic clustering revealed meaningful subgroups related to survivorship experiences, including discussions of protein powders, cancer-related weight loss, breast cancer and estrogen receptor status, and vitamins for future cancer prevention.

Second, sentiment-stratified analysis revealed that reviews with lower sentiment scores more often focused on cancer-related risks (eg, toxic ingredients and product harms), while those with higher scores more often highlighted perceived benefits of supplements and other supportive products for managing cancer-related symptoms (Figures S1-S6 in Multimedia Appendix 4). These patterns provide insights into how survivors interpret and evaluate complementary therapies in relation to their health and recovery.

Third, associations between specific cancer types (identified via the rule-based dictionary method), and symptoms (identified using zero-shot LLM methods) surfaced detailed symptom management experiences, including pain, fatigue, and gastrointestinal symptoms. Notably, the top 15 symptoms included in these reviews reflect common survivorship challenges [40,41], with pain being the most frequent. Fourth, while the zero-shot LLM was not formally evaluated in this content analysis, it was effective in identifying symptom-related language at scale, which enabled exploratory symptom mapping across cancer types. These findings illustrate how publicly available consumer data can offer valuable insight into the lived experiences of survivors and their efforts to manage persistent symptoms using accessible, over-the-counter, or complementary therapies.

Beyond content analysis, our key contributions include the development of a manually annotated dataset with 159 reviews and baseline NLP models for NER and text classification. This resource was intentionally designed to capture nuanced mentions of cancer types (eg, “cancer in his bone”) and survivor-reported outcomes, laying the groundwork for future applications in survivorship research. These annotations provide a foundation for fine-grained analysis of survivor narratives and outcomes related to self-management of cancer and treatment-related side effects.

The baseline models demonstrated promising performance. The bert-base-cased model achieved the highest weighted average *F*_1_-score for NER, while gpt4-1106-preview-chat achieved the highest *F*_1_-scores across all text classification tasks. These results suggest that LLMs, while currently limited in NER performance [42,43], are highly effective for text classification. For instance, even in the limited Harmful outcome category (n=16), GPT-4 was able to generalize and achieve an *F*_1_-score of 0.6667 in the zero-shot setting, compared with an *F*_1_-score of 0 for fine-tuned BERT/Bio_ClinicalBERT models. This highlights the potential of LLMs for use in survivorship-related classification tasks where annotated data may be limited. To address the low performance of the NER task, data augmentation through synthetic data generation or fine-tuning models with a larger training dataset can be used.

### Limitations

Our study has several limitations. First, the sentiment analysis component is constrained by the domain dependence of existing pretrained models [44]. While many general-purpose language models can classify sentiment, few are trained specifically on health or cancer-related content. To enable a high-level analysis of emotional tone, we used an existing sentiment model, which achieved 67% exact match accuracy between predicted sentiment and the number of stars assigned to product reviews containing cancer mentions. However, this approach occasionally produced mismatches. For instance, the sentence “My husband took this for early stage CLL and after 9 months is in remission.” was assigned a sentiment score of 1 (negative), despite clearly expressing a positive outcome. This misalignment reflects the complexity of interpreting sentiment in survivorship contexts, where emotional tone may be influenced by both product experience and the reviewer’s cancer journey. As such, sentiment scores may not consistently reflect product efficacy or survivor satisfaction. In addition, consumer reviews are inherently subjective and may reflect social influence biases (eg, other reviews) [45], or may come from users who are not representative of the broader survivor population [46]. Despite these limitations, sentiment analysis helped highlight broad differences in topics across emotional tone. In future work, we plan to fine-tune domain-specific sentiment models trained on health-related and survivorship-specific data to improve classification accuracy and interpretability.

Second, although the overall IAA was high (*F*_1_=0.86), the IAA for the “Harmful outcomes” category was considerably lower (*F*_1_=0.63). This is likely attributable to the small number of annotated instances (n=16), which may have contributed to reduced consistency. However, given the clinical and survivorship importance of identifying adverse outcomes, this remains a critical category. Future annotation efforts will involve expanded training, guideline refinement, and targeted oversampling of underrepresented classes to improve reliability.

Third, the dataset used for this study includes Amazon reviews posted between May 1996 and July 2014. Consumer behaviors, complementary therapy trends, and survivorship care practices have likely evolved in the past decade. This temporal limitation may restrict the contemporary relevance of some findings, particularly in light of recent shifts toward integrative oncology and growing digital health engagement among survivors. Fourth, our manually annotated dataset comprises only 159 reviews. While this proof-of-concept sample enabled initial model development and feasibility testing, the limited sample size constrains the generalizability and robustness of the resulting models. Ongoing annotation work will expand the dataset, with careful attention to balancing reviews across outcome types and sentiment categories to support more comprehensive model training.

Notably, a new version of the Amazon review dataset—spanning May 1996 to Sep 2023—has recently been released and is 245.2% larger than the version used in this study [47]. Future work will leverage the expanded dataset to scale annotation efforts, develop more robust models, and generate updated insights into cancer symptom management and complementary therapy use among cancer survivors. These analyses could also support regulatory efforts and health care interventions by highlighting potential product risks and unmet survivor needs reflected in real-world consumer narratives.

### Conclusion

Our results demonstrate the potential of Amazon consumer reviews as a novel data source for identifying persistent symptoms, concerns, and self-management strategies among cancer survivors. We presented the design and implementation of a publicly accessible, manually annotated corpus available through the OHNLP GitHub focused on cancer type, symptoms, and symptom management outcomes. This corpus, along with the baseline NLP models developed for named entity recognition and text classification, lays the groundwork for future methodological advancements in cancer survivorship research. Importantly, insights derived from this study could be evaluated in relation to established clinical guidelines for symptom management in cancer survivorship care (eg, American Society of Clinical Oncologists and National Comprehensive Cancer Network). Such comparisons may help validate survivor-reported outcomes, reveal novel survivor concerns not routinely captured in clinical care settings, and inform the development of more patient-centered care models. By revealing the feasibility of using consumer-generated data for mining survivorship-related experiences, this study offers a promising foundation for future research and argumentation analysis aimed at improving long-term outcomes and support for cancer survivors.

## Supplementary material

10.2196/71102 Multimedia Appendix 1 Amazon review - annotation guidelines.

10.2196/71102 Multimedia Appendix 2 Tables showing schema of the annotated labels, distribution of sentiment scores across the sentences with cancer mentions, number of sentences corresponding to each cluster, and inter-annotator agreements.

10.2196/71102 Multimedia Appendix 3 Large language model prompting instruction.

10.2196/71102 Multimedia Appendix 4 Figures depicting hierarchical clustering and topic modeling.

## Abbreviations

ASIN: Amazon Standard Identification Number
LLM: large language model
NER: named entity recognition
NLP: natural language processing
OHNLP: open health natural language processing
SFT: supervised fine-tuning
UMAP: uniform manifold approximation and projection
IAA: inter-annotator agreement
