# Supplementary material for: Understanding Cancer Survivorship Care Needs Using Amazon Reviews: Content Analysis, Algorithm Development, and Validation Study
Source: JMIR Cancer. 2025 Sep 23;11:e71102. doi: 10.2196/71102 (PMC12456872; doi:10.2196/71102)
Supplement: Multimedia Appendix 1 [file cancer-v11-e71102-s001.docx]

**Amazon Review - Annotation Guidelines**

Contents

1 Concepts 3

1.1 Cancer types 3

1.2 Indicated symptoms 4

1.3 Harmful outcome 5

1.4 Favorable outcome 5

1.5 Product 6

2 Annotation Tool 6

# Concepts

## Cancer types

If there is a mention of detailed cancer types, such as “breast cancer”, annotate “breast cancer” instead of “cancer”. Note, some cancer may not appear as “cancer”, for example, leukemia, lymphoma, melanoma.

*Class includes human or pet.*

*Certainty includes Positive, Negative, Hypothetical and Possible.*

**Example: Cancer_type, Hypothetical, Human**

*
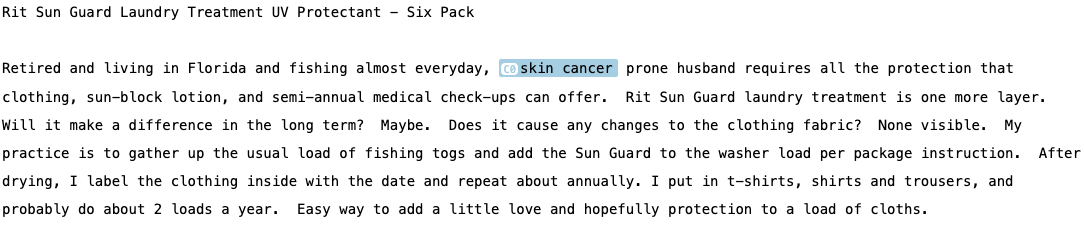
*

**Example: Cancer_type, Positive, Human**

*
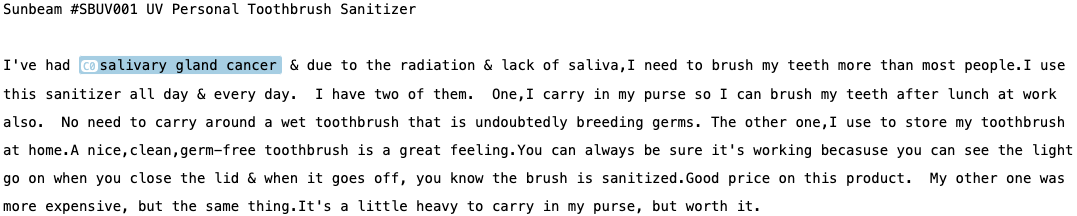
*

**Example: Cancer_type, Positive, Pet**


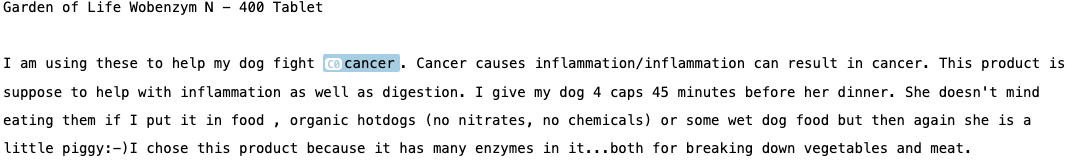


## Indicated symptoms

The symptoms that the Amazon product was used for.

*Class includes cancer related or other.*

*Certainty includes positive, negative, hypothetical, or possible.*

**Example: Indicated_symptom, Cancer_related, Positive**

*
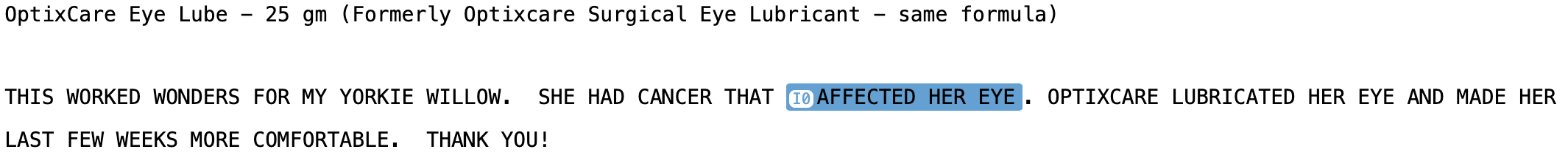
*

**Example: Indicated_symptom, Other, Positive**

*
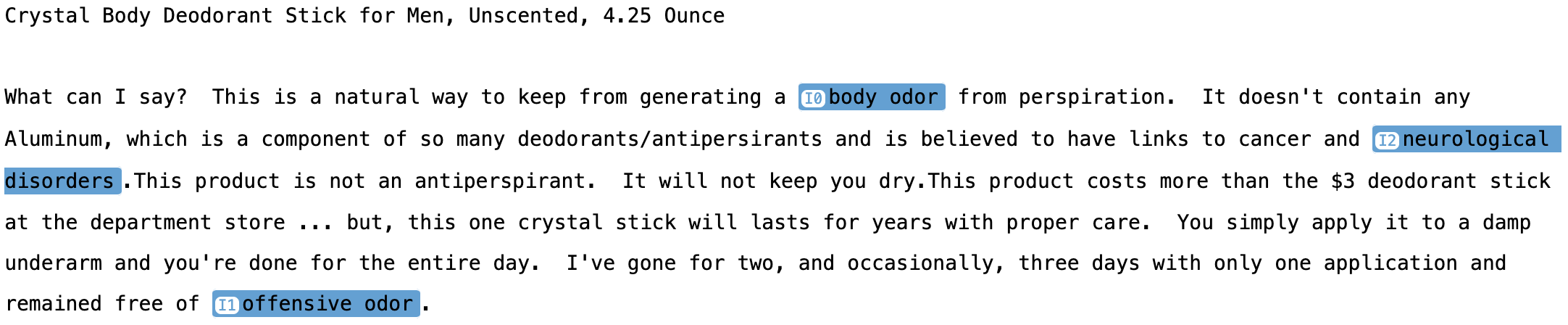
*

**Example: Indicated_symptom, Cancer_related, Hypothetical**

*
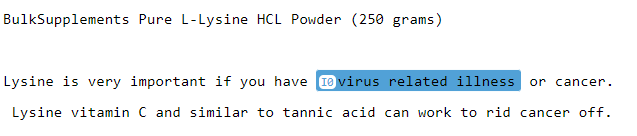
*

**Example: Indicated_symptom, Other, Negative**

*
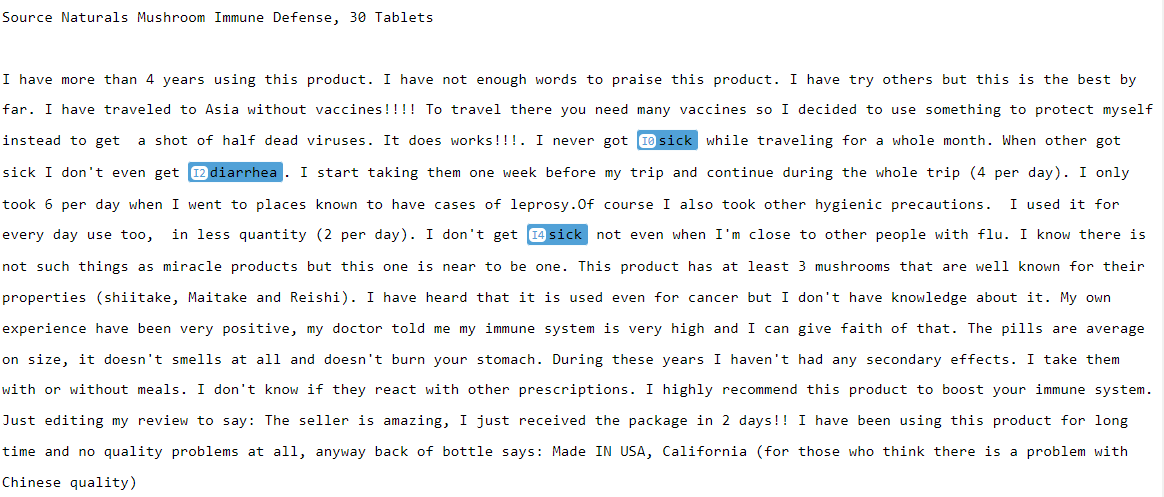
*

## Harmful outcome

Adverse or harmful outcome after using the product.

*Class includes cancer related or other.*

*Certainty includes positive, negative, hypothetical, or possible.*

**Example: Harmful_outcome, Cancer_related, Positive**

*
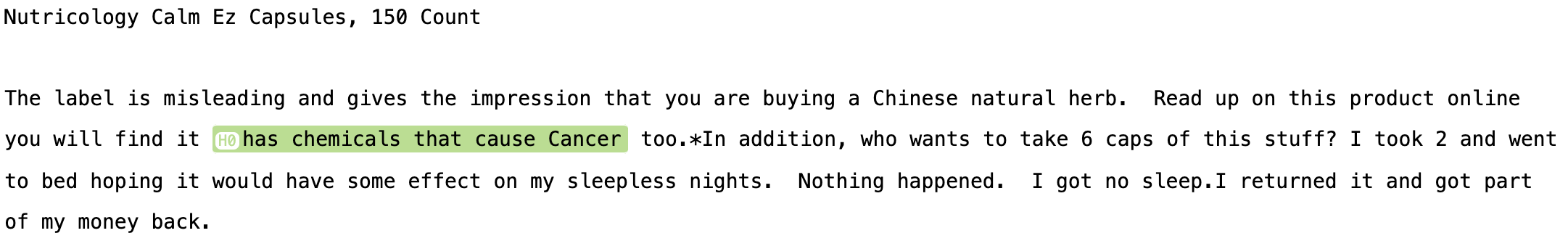
*

**Example: Harmful_outcome, Cancer_related, Hypothetical**


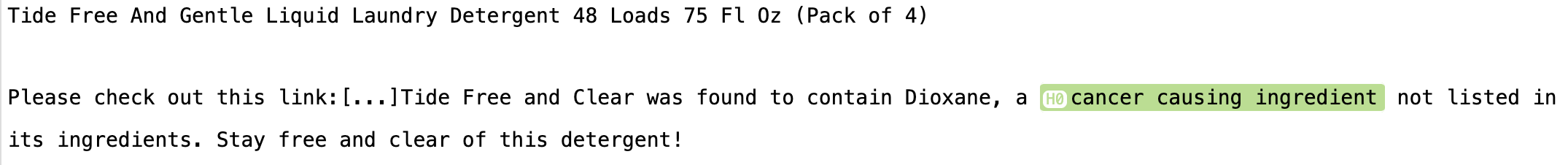


## Favorable outcome

Beneficial or favorable outcome after using the product.

*Class includes cancer related or other.*

*Certainty includes positive, negative, hypothetical, or possible.*

**Example: Favorable outcome, Positive**

*
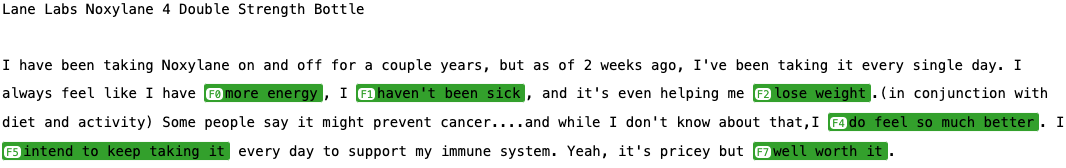
*

**Example: Favorable outcome, Hypothetical**

*
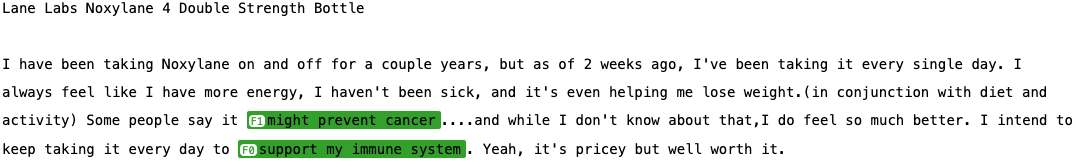
*

**Example: Favorable outcome, Other, Positive/Negative**

*
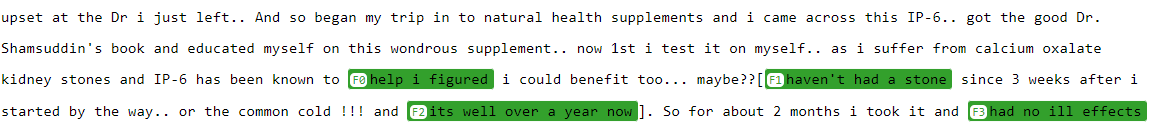
*

## Product

The mention of the product name or the co-reference of the product such as “this”.

*Type includes Itself or other.*

**Example: Product, Itself**

*
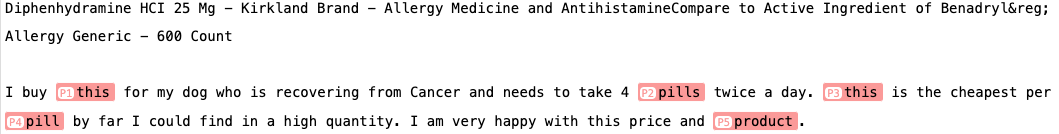
*

**Example: Product, Other**


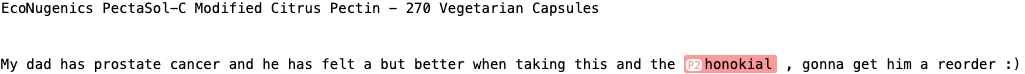


# Annotation Tool

We used MedTator in this annotation task.
