# Supplementary material for: Understanding Cancer Survivorship Care Needs Using Amazon Reviews: Content Analysis, Algorithm Development, and Validation Study
Source: JMIR Cancer. 2025 Sep 23;11:e71102. doi: 10.2196/71102 (PMC12456872; doi:10.2196/71102)
Supplement: Multimedia Appendix 2 [file cancer-v11-e71102-s002.docx]

**Supplementary Table 1. Schema of the annotated labels.**

| **Concepts** | **Class/Type** | | **Certainty** | | | |
| --- | --- | --- | --- | --- | --- | --- |
| Cancer_type | Human | Pet | Positive | Negative | Hypothetical | Possible |
| Indicated_symptom | Cancer_related | Other | Positive | Negative | Hypothetical | Possible |
| Harmful_outcome | Cancer_related | Other | Positive | Negative | Hypothetical | Possible |
| Favorable_outcome | Cancer_related | Other | Positive | Negative | Hypothetical | Possible |
| Product | Itself | Other | NA | NA | NA | NA |

**Supplementary Table 2. Distribution of sentiment scores across the sentences with cancer mentions.**

| Sentiment score | Number of sentences |
| --- | --- |
| 1 | 1048 |
| 2 | 788 |
| 3 | 488 |
| 4 | 932 |
| 5 | 1447 |

**Supplementary Table 3. Number of sentences corresponding to each cluster.** The “Error” class means the sentences cannot be labeled by GPT-4o.

| Cluster | Number of sentences |
| --- | --- |
| Cancer Support, Symptoms & General Health | 963 |
| Cancer Prevention & Supplementation | 617 |
| Cancer Survivorship & Treatment Journeys | 612 |
| Cancer Research & Alternative Treatments | 349 |
| Environmental &Chemical Cancer Risks | 164 |
| Scientific Studies & Genetic Factors | 126 |
| General Cancer Concerns & Alternative Health | 79 |
| Error | 3 |

**Supplementary Table 4. Inter-annotator agreements**

| Concept | F1 |
| --- | --- |
| Overall | 0.86 |
| Cancer_type | 0.97 |
| Indicated_symptom | 0.81 |
| Harmful_outcome | 0.63 |
| Favorable_outcome | 0.70 |
| Product | 0.91 |
