# Supplementary material for: Understanding Cancer Survivorship Care Needs Using Amazon Reviews: Content Analysis, Algorithm Development, and Validation Study
Source: JMIR Cancer. 2025 Sep 23;11:e71102. doi: 10.2196/71102 (PMC12456872; doi:10.2196/71102)
Supplement: Multimedia Appendix 3 [file cancer-v11-e71102-s003.docx]

**LLM prompting instruction**

### Instructions for labeling sentences as one of the seven topics derived from hierarchical clustering based on its content.

def instructions_text():

    return """

    Given an input sentence, classify it into one of the following seven topics based on its content. The topics are:

    1. General Cancer Concerns & Alternative Health

    2. Environmental & Chemical Cancer Risks

    3. Cancer Research & Alternative Treatments

    4. Scientific Studies & Genetic Factors

    5. Cancer Survivorship & Treatment Journeys

    6. Cancer Prevention & Supplementation

    7. Cancer Support, Symptoms & General Health

    Your task is to output only the corresponding class number (1 to 7) that best fits the sentence.

    """

### Instruction for NER

Your role involves identifying named entities in the text and applying the BIO labeling scheme. Start by marking the beginning of a related phrase with B (Begin), and then continue with I (Inner) for the subsequent words within that phrase. Utilize the following labels to classify each entity: Cancer_type: If the entity represents a type of cancer. Indicated_symptom: If the entity represents a symptom. Product: If the entity refers to the mention of the product name or the co-reference of the product such as “this”. O: If the entity does not fit into any of the above categories. So the label should be one of the following: [B-Cancer_type, I-Cancer_type, B-Indicated_symptom, I-Indicated_symptom, B-Product, I-Product, O].

For each input token provided, generate a corresponding label. Ensure that each output is presented on a separate line, in the format of [input token : label]

### Input: {sent}

### Output:

### Instruction for text classification

You are an AI model designed to classify sentences from Amazon customer reviews about cancer-related products. Each input sentence will be classified into one of three categories: "HARMFUL", "FAVORABLE", or "AMBIGUOUS". Your output should be a numerical label according to the following mapping: "HARMFUL" is 0, "FAVORABLE" is 1, and "AMBIGUOUS" is 2.

Task:

Given a sentence from an Amazon customer review about a cancer-related product, analyze the sentiment and content of the sentence to determine its classification. Use the context and tone of the sentence to make your determination. Output the appropriate numerical label for each sentence based on the provided mapping.

### Input: {sent}

### Output:
