# Supplementary material for: Understanding Cancer Survivorship Care Needs Using Amazon Reviews: Content Analysis, Algorithm Development, and Validation Study
Source: JMIR Cancer. 2025 Sep 23;11:e71102. doi: 10.2196/71102 (PMC12456872; doi:10.2196/71102)
Supplement: Multimedia Appendix 4 [file cancer-v11-e71102-s004.docx]

**
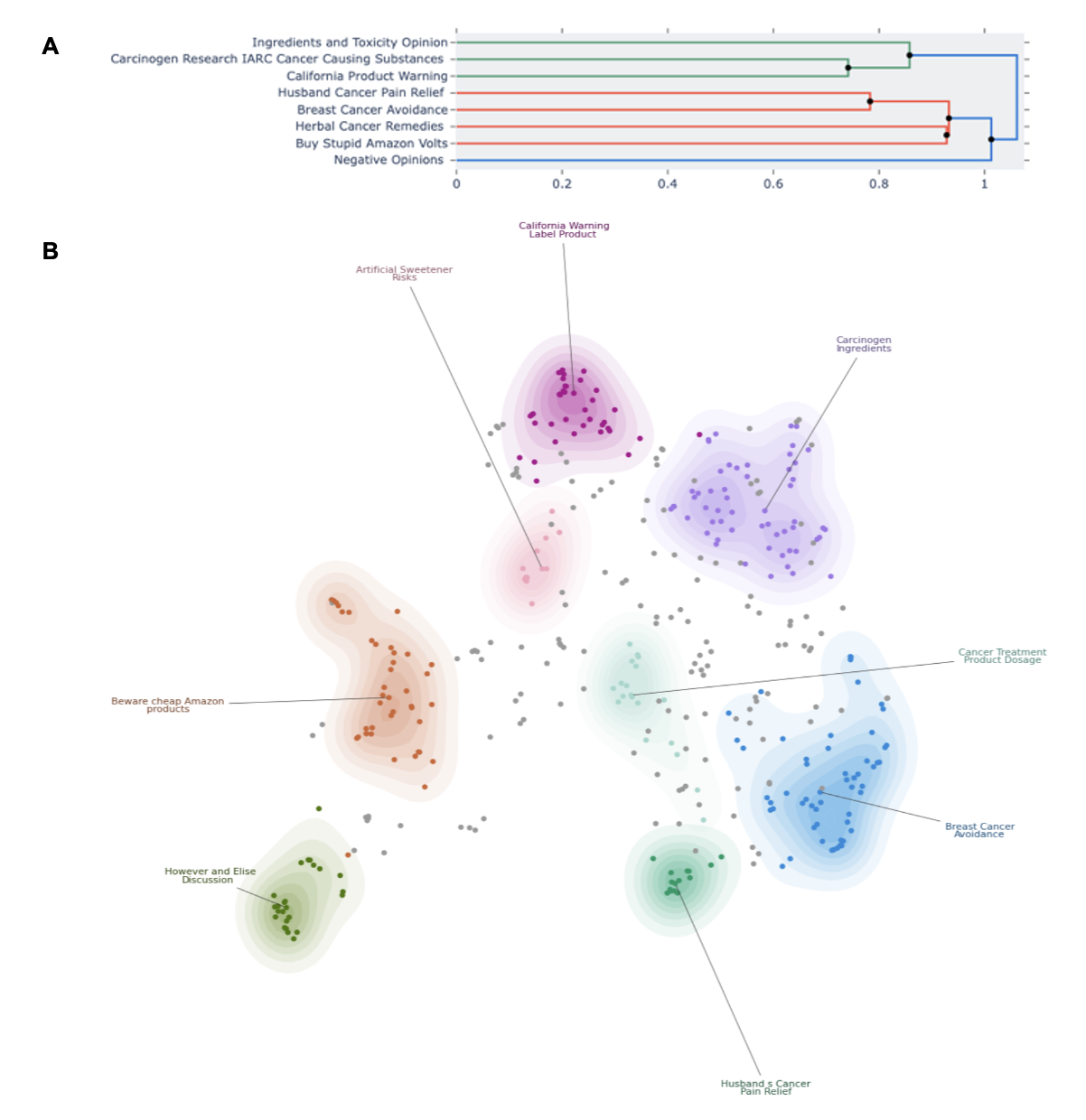
Supplementary Figure 1. Hierarchical clustering (A) and topic modeling (B) based on sentences with semantic score 1.**

**
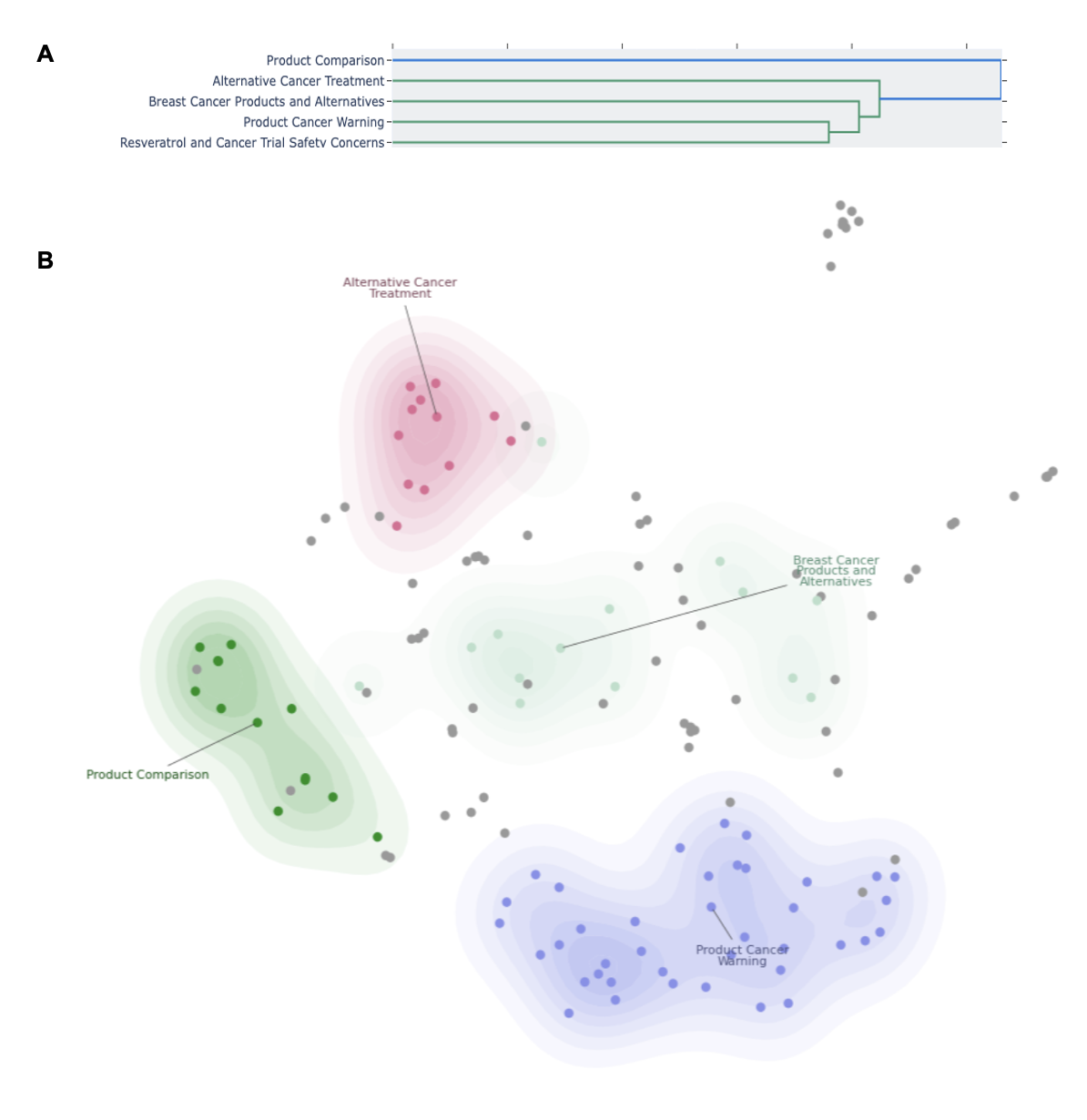
**

**Supplementary Figure 2. Hierarchical clustering and topic modeling based on sentences with semantic score 2.**

**
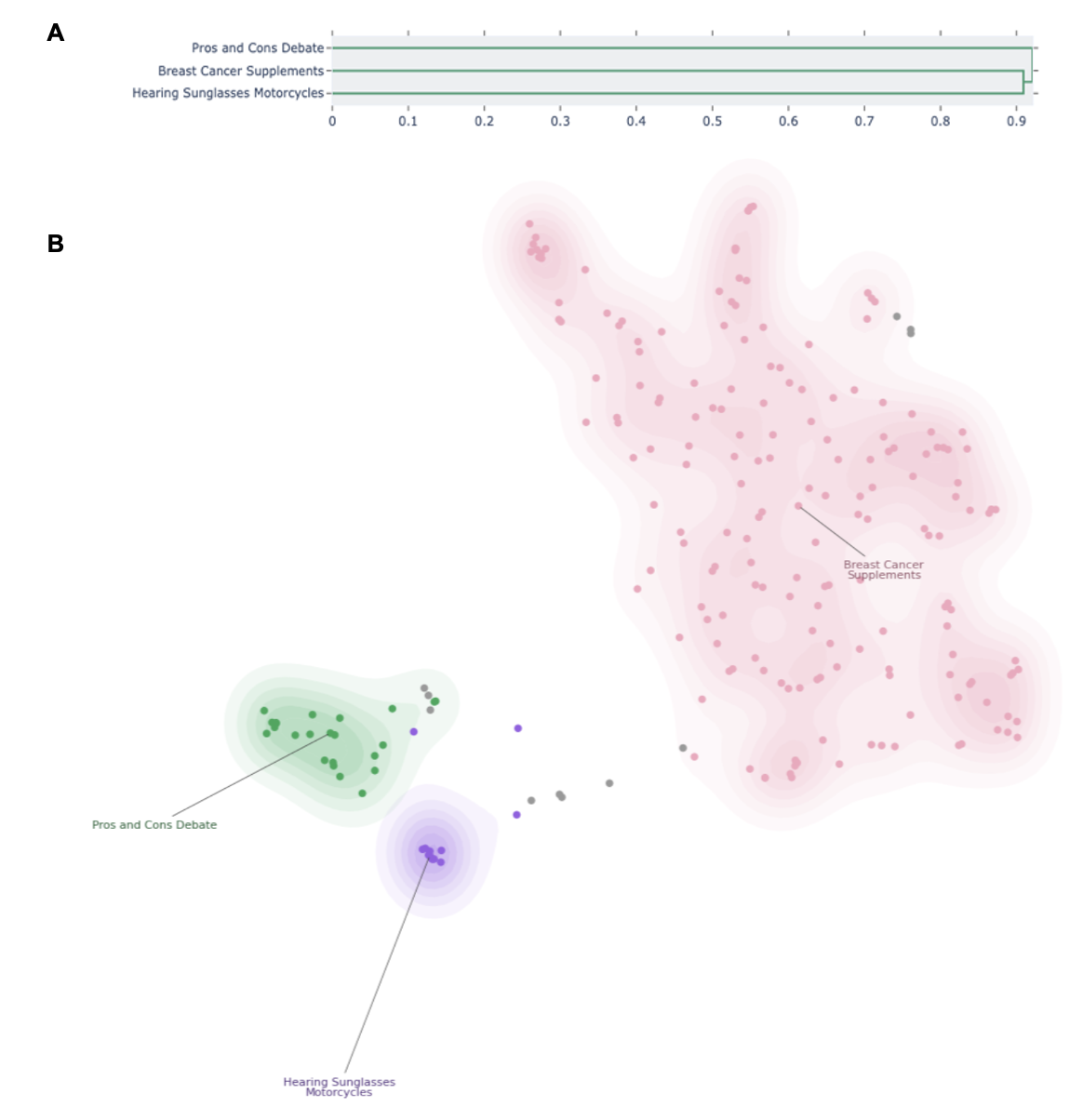
Supplementary Figure 3. Hierarchical clustering and topic modeling based on sentences with semantic score 3.**

**
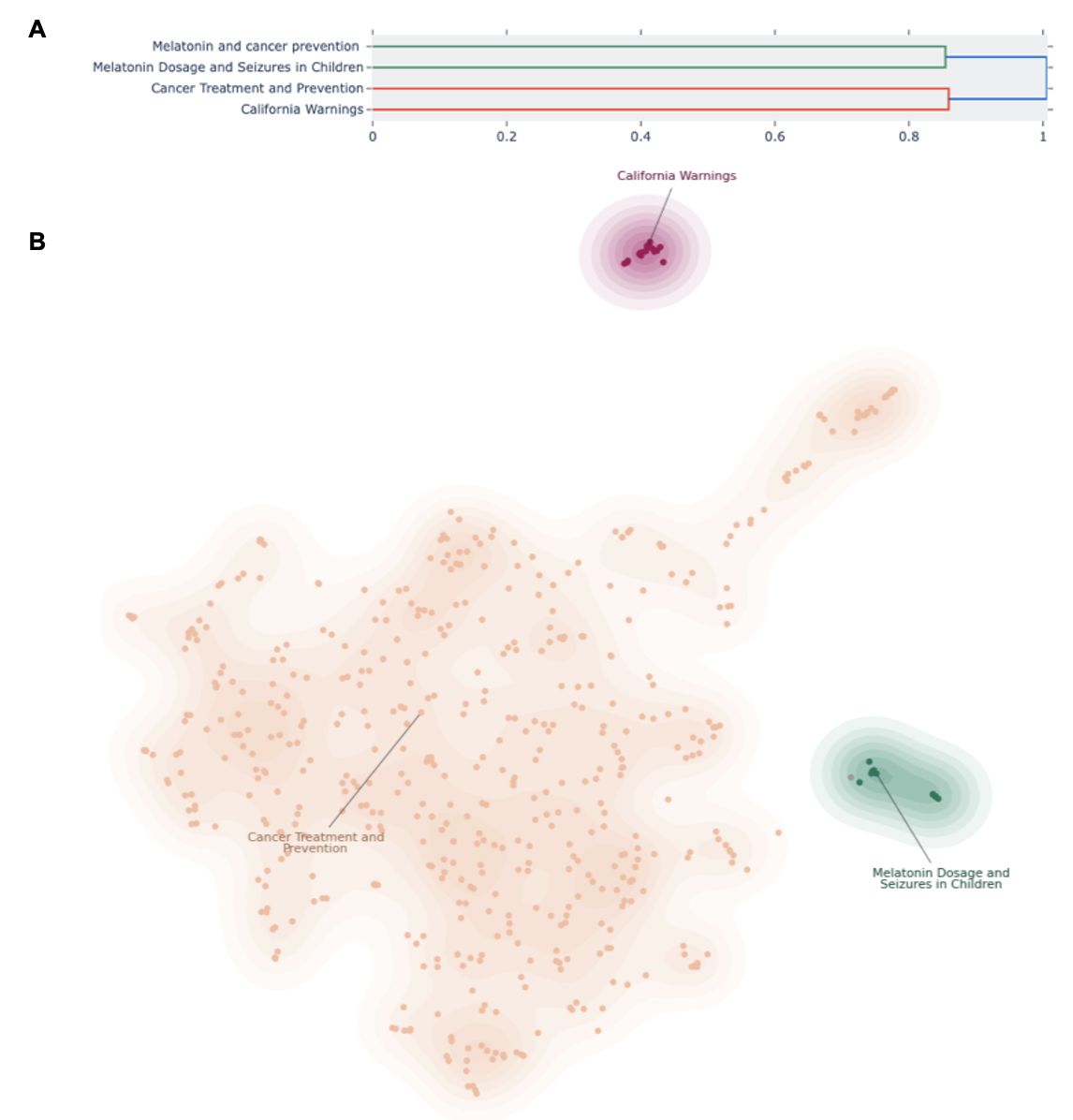
**

**Supplementary Figure 4. Hierarchical clustering and topic modeling based on sentences with semantic score 4.**

**
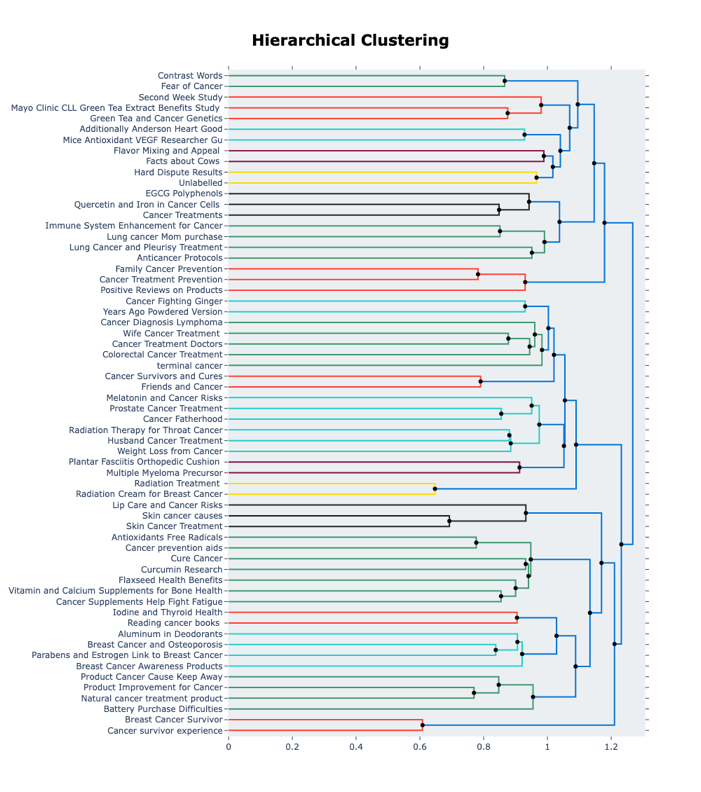
**

**Supplementary Figure 5. Hierarchical clustering based on sentences with semantic score 5.**

**
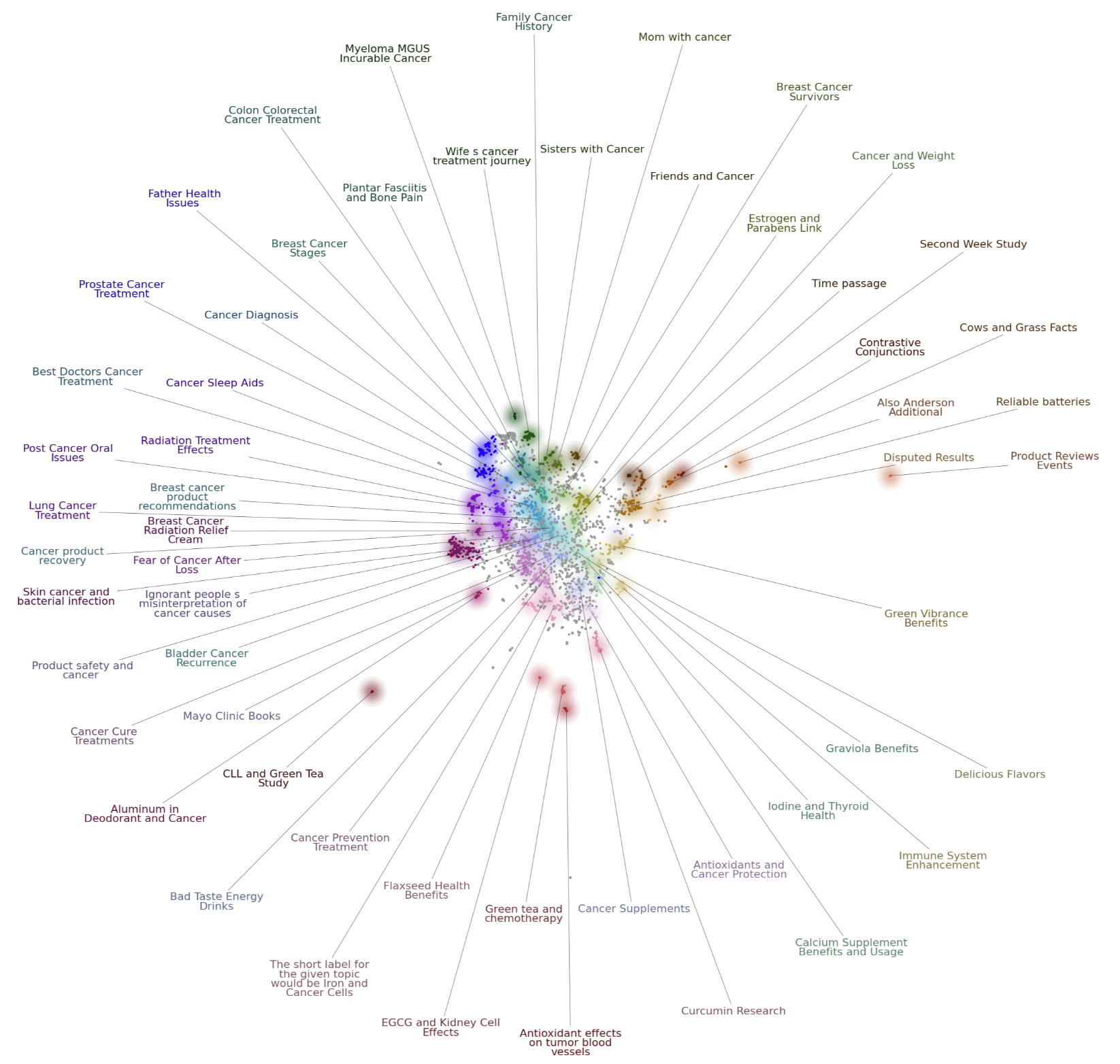
**

**Supplementary Figure 6. Topic modeling based on sentences with semantic score 5.**
